# Measurement-Based Care to Enhance Antidepressant Treatment Outcomes in Major Depressive Disorder: A Randomized Clinical Trial

**DOI:** 10.1001/jamanetworkopen.2025.29427

**Published:** 2025-09-02

**Authors:** Muhammad Ishrat Husain, Zahra Nigah, Sami Ul Haque Ansari, Ameer B. Khoso, Tayyeba Kiran, Madeha Umer, Moin A. Ansari, Moti Ram Bhatia, Sylvia A. Khan, Muhammad Omair Husain, Abdul Ghafoor Malik, Haider Ali Naqvi, Altaf Qadir, Aatir H. Rajput, Mohsin Saqib, Muhammad Ilyas, Mujeeb Ullah Khan Doutani, Silsila Sherzad, K. M. Sajjad Siddiqui, Zona Tahir, Wei Wang, Nusrat Husain, Nasim Chaudhry, Imran Bashir Chaudhry, Benoit H. Mulsant

**Affiliations:** 1 Campbell Family Mental Health Research Institute, Centre for Addiction and Mental Health, Toronto, Ontario, Canada; 2 Department of Psychiatry, Temerty Faculty of Medicine, University of Toronto, Toronto, Ontario, Canada; 3 Pakistan Institute of Living and Learning, Karachi, Pakistan; 4 Liaqat University of Medical & Health Sciences, Cowasjee Jehangir Institute of Psychiatry, Hyderabad, Pakistan; 5 Department of Psychiatry, Peoples University of Medical and Health Sciences, Shaheed Benazirabad, Pakistan; 6 Department of Psychiatry, North West General Hospital, Peshawar, Pakistan; 7 Department of Psychiatry, Benazir Bhutto Hospital, Rawalpindi, Pakistan; 8 Department of Psychiatry, Dow University of Health Sciences, Karachi, Pakistan; 9 Pakistan Institute of Living and Learning, Lahore, Pakistan; 10 Baluchistan Institute of Psychiatry and Behavioral Sciences, Quetta, Pakistan; 11 Department of Psychiatry, National Psychiatric Hospital, Multan, Pakistan; 12 Department of Psychological Clinical Science, University of Toronto Scarborough, Toronto, Ontario, Canada; 13 Department of Biostatistics and Data Science, University of South Florida, Tampa; 14 Division of Psychology and Mental Health, School of Health Sciences, University of Manchester, Manchester, United Kingdom; 15 Mersey Care National Health Service Foundation Trust, Liverpool, United Kingdom; 16 Department of Psychiatry, Ziauddin University, Karachi, Pakistan

## Abstract

**Importance:**

Measurement-based care (MBC) guides clinical decisions through structured monitoring of symptoms and adverse effects. Although MBC has been associated with improved outcomes in major depressive disorder (MDD), its effectiveness in low- and middle-income countries (LMICs) remains understudied.

**Objective:**

To assess whether MBC accelerates the resolution of depressive symptoms compared with standard care among adults with MDD in Pakistan.

**Design, Setting, and Participants:**

This multicenter, assessor-blinded, parallel-arm randomized clinical trial was conducted in Pakistan from September 2022 to January 2024, with 24 weeks of follow-up. Adults diagnosed with nonpsychotic MDD were recruited from psychiatric hospitals and primary care centers in 7 Pakistani cities (Karachi, Lahore, Rawalpindi, Hyderabad, Peshawar, Multan, and Quetta). Participants were randomized 1:1 to MBC or standard care. Intention-to-treat analyses were conducted.

**Interventions:**

By design, pharmacotherapy was limited to paroxetine or mirtazapine in both MBC or standard care groups. The MBC group completed the 16-item Quick Inventory of Depressive Symptomatology–Self-Report and the Frequency, Intensity, and Burden of Side Effects Rating Scale at each visit (baseline and weeks 2, 4, 8, 12, and 24). Scores from these instruments informed antidepressant dose adjustments or switch. The standard care group received treatment based on clinician judgment and did not undergo repeated clinical measurements.

**Main Outcomes and Measures:**

Primary outcomes were time to response (defined as ≥50% reduction in the 17-item Hamilton Depression Rating Scale [HDRS-17]; range: 0-52, with the highest score indicating severe depression) and time to remission (defined as HDRS-17 score of ≤7) within the 24-week follow-up period. Secondary outcomes included changes in HDRS-17 scores and rates of adverse effects or treatment discontinuation.

**Results:**

A total of 154 adults (mean [SD] age, 34.5 [10.5] years; 105 females [68.2%]) were randomized. Median (IQR) time to response was faster with MBC than with standard care (2 [2-4] weeks vs 4 [2-12] weeks); similarly, median (IQR) time to remission was faster for MBC vs standard care (4 [4-8] weeks vs 8 weeks [2 weeks to no remission] weeks). At week 24, there were no significant differences in rates of response or remission between groups. After week 24, reduction in mean HDRS-17 scores was significantly but modestly greater in the MBC group than in the standard care group (−18.1 [95% CI, 16.4-19.6] points vs −17.0 [95% CI, 15.6-18.5] points; *t*_129_ = 0.71; *P* < .001). No differences were observed in other secondary outcomes.

**Conclusions and Relevance:**

This trial of adults with MDD found that MBC led to faster time to response and time to remission than standard care in low-resource settings. Future studies need to confirm the clinical effectiveness of MBC and assess its cost-effectiveness in LMICs.

**Trial Registration:**

ClinicalTrials.gov Identifier: NCT05431374

## Key Points

**Question:**

Does measurement-based care (MBC) reduce time to response and time to remission compared with standard care for major depressive disorder (MDD) in a low- and middle-income country such as Pakistan?

**Findings:**

In a randomized clinical trial of 154 adults with nonpsychotic MDD, the time to response and time to remission were half as long among those who received MBC compared with those who received standard care.

**Meaning:**

Findings of this trial indicate that MBC may improve treatment outcomes for MDD in resource-limited settings such as Pakistan.

## Teaser

This randomized clinical trial evaluates whether adults receiving measurement-based care vs standard care for major depressive disorder have faster response and remission times.

## Introduction

Major depressive disorder (MDD) is a leading cause of disability across populations and settings.^1^ The global incidence of MDD has increased over the past 3 decades,^2^ with the current prevalence being higher in low- and middle-income countries (LMICs) than in high-income countries (HICs).^3^ Despite access to effective pharmacotherapy and psychotherapy in HICs, only a third of patients with MDD achieve remission.^4^ Inadequate antidepressant dose and duration of antidepressant trials have been identified as the main causes for poor outcomes.^5^

Over the past decade, measurement-based care (MBC) has been proposed as a solution to this challenge. MBC is a simple intervention for use of antidepressants that is based on regular monitoring and applies validated clinical instruments to guide treatment decisions.^6^ While there is evidence of the effectiveness of MBC in the management of MDD in HICs^7^ and some upper middle–income countries, such as China,^8^ we are not aware of any evaluations of MBC in LMICs. Given the high prevalence of MDD and the lack of access to specialized psychiatric services in these settings, MBC may offer a scalable approach to enhance outcomes for patients with MDD in LMICs. In this context, we conducted a randomized clinical trial (RCT) comparing MBC vs standard care for MDD in Pakistan. Time to response and time to remission were chosen as the primary outcomes to assess whether MBC accelerates the resolution of depressive symptoms among adults with MDD as this is important in low-resource settings (eg, LMICs), where access to care and treatment options are limited. We hypothesized that MBC would lead to faster antidepressant response and symptom remission than standard care.

## Methods

This multicenter, assessor-blinded, parallel-arm RCT was conducted in Pakistan between September 2022 and January 2024, replicating the design of a trial by Guo et al^8^ conducted in Beijing, China. Antidepressants were fixed to paroxetine or mirtazapine in both treatment groups to minimize heterogeneity in outcomes due to variability in response to and tolerability of specific antidepressants^9^ and to allow the isolation of the effects of MBC. Doses were converted to amitriptyline equivalents (ie, 10-mg paroxetine or 15-mg mirtazapine = 50-mg amitriptyline). Pakistan’s National Bioethics Committee approved this trial; the trial protocol is available in Supplement 1. All participants provided written informed consent. We followed the Consolidated Standards of Reporting Trials (CONSORT) reporting guideline.^10^

### Participants and Settings

Participants were recruited from outpatient departments in general and psychiatric hospitals or primary care clinics and Basic Health Units in 7 major cities in Pakistan: Karachi (23 million), Lahore (10 million), Rawalpindi (3 million), Hyderabad (2 million), Peshawar (2 million), Multan (1.8 million), and Quetta (1 million). These urban locations represent a combined population of more than 42 million people (out of 236 million Pakistanis) and serve as hubs for both public and private mental health care.

Participants were adult outpatients aged 18 to 65 years with nonpsychotic MDD (diagnosed by their treating physicians and confirmed using a checklist based on the *Diagnostic and Statistical Manual of Mental Disorders, Fifth Edition* [*DSM-5*] criteria at screening), a score of 18 or higher (indicating moderate to severe depression) on the 17-item Hamilton Depression Rating (HDRS-17), and the ability to speak English or Urdu fluently and to provide written informed consent. Potential participants were excluded if they had a lifetime diagnosis of bipolar, psychotic, obsessive-compulsive, eating, or substance use (excluding nicotine) disorder based on the *DSM-5* criteria; a history of a lack of response or intolerance to paroxetine or mirtazapine; a suicide attempt during the current depressive episode; any physical condition contraindicating the use of paroxetine or mirtazapine; or were pregnant or breastfeeding.

### Sample Size

The target sample size replicated the sample size of the Guo et al^8^ trial. Anticipating a 20% dropout rate, we aimed to randomize 150 participants to obtain 120 completers. Given the lack of robust data on expected differences in time to remission in LMICs, as Guo et al^8^ used, we relied on estimates of remission rates to calculate the power based on the sample size. With a difference of 45% in remission rates (ie, 74% MBC vs 29% standard care in the Guo et al^8^ trial), 120 completers provided greater than 99% power at α = .05 to detect a difference in remission rates. Even with a smaller effect size of 18% (ie, 59% vs 41% in the Guo et al^8^ trial), power remained greater than 80%.^11^

### Randomization and Masking

**Figure 1.**
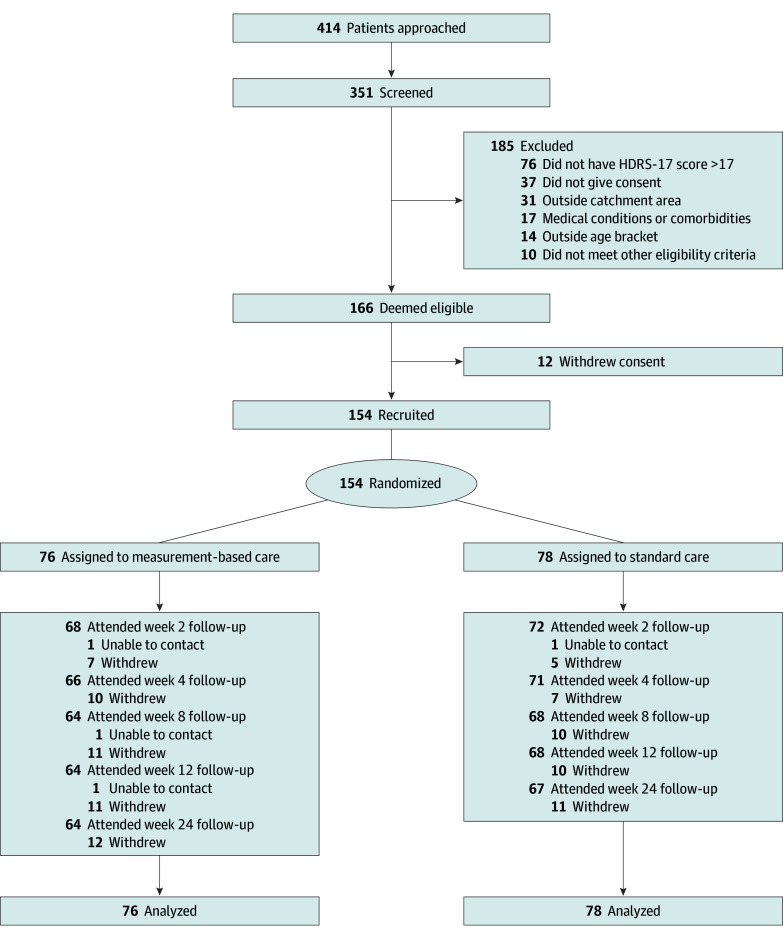
Participant Flow Diagram HDRS-17 indicates the 17-item Hamilton Depression Rating Scale (range: 0-52, with the highest score indicating severe depression).

Participants were randomized 1:1 to MBC or standard care (Figure 1) using 14 computer-generated lists with random permuted blocks, stratified by site and self-reported sex at birth. Given the nature of the intervention, physicians and participants were not masked to treatment allocation, but all of the outcome assessors were. Participants were instructed not to disclose their treatment group to outcome assessors, and new assessors were assigned if unintentional unblinding occurred.

### Interventions and Procedures

After providing informed consent, participants entered a 1-week washout phase to minimize the effects of psychotropic medications previously taken. After the washout week, the participants were assigned randomly to either the MBC group or the standard care group. Regardless of their treatment group, participants received either open-label paroxetine (10-60 mg/d) or open-label mirtazapine (7.5-45 mg/d). All patients were followed up for 24 weeks after randomization, with research assessments at baseline and weeks 2, 4, 8, 12, and 24.

Starting doses were permitted to be lower than those used in the Guo et al^8^ trial in response to feedback from the Trial Steering Committee about patient preference for slower medication titration in the local population. In both treatment groups, the treating physician (ie, general practitioner or psychiatrist) was responsible for decisions about the prescription of paroxetine or mirtazapine and dose adjustments. One medication change between paroxetine and mirtazapine was permitted. Short-acting benzodiazepines could be prescribed occasionally for agitation, anxiety, or insomnia. Changes in antidepressant or concomitant treatment (either pharmacotherapy or psychotherapy) were recorded by the research staff at each visit.

#### MBC Intervention

At every outpatient visit, participant symptoms and adverse effects were recorded through the 16-item Quick Inventory of Depressive Symptomatology–Self-Report (QIDS-SR16)^12^ and the Frequency, Intensity, and Burden of Side Effects Rating (FIBSER) Scale.^13^ The QIDS-SR16 was used to measure depressive symptoms in the past week; it has a score range of 0 to 27, with higher scores indicating higher severity.^12^ An elevated QIDS-SR16 total score was indicative of current treatment being unhelpful and requiring an increase of dose or a switch in medication depending on the week of treatment.^8^ The FIBSER Scale was completed to evaluate antidepressants’ adverse effects, their frequency, intensity, and burden.^13,14^ A FIBSER Scale total score higher than 4 on a scale of 0 to 18 was indicative of current treatment being poorly tolerated and requiring a change of dose or a switch in medication.^8^

Participant responses on these self-reported measures informed and guided the physicians’ decisions. Following a stepwise approach, a treatment protocol outlined recommended dose adjustments or medication switches needed to reduce depressive symptoms (reported on the QIDS-SR16) or to mitigate adverse effects (reported on the FIBSER Scale) (eTable 1 in Supplement 2). Treating physicians underwent a 2-day training on this MBC protocol. In addition, a coordinator was designated at each study site to serve as an MBC coordinator and to facilitate adherence with the MBC treatment protocol. After each clinical visit, this coordinator compared the physician’s clinical notes with the MBC guidelines. When deviations were identified, the physician was promptly notified and encouraged to adjust the treatment accordingly.

#### Standard Care Control

Local medical, psychiatric, and general practitioner services provided routine care according to their clinical judgment and available resources. Standard care was directed by the participant’s treating physician based solely on clinical needs (ie, clinical measures were not obtained, and treatment protocol was not established). Research staff recorded the nature and intensity of standard care delivered to each participant. In current practice in Pakistan, standard care of MDD largely consists of pharmacotherapy, and patients with MDD are not routinely referred for any psychological therapies.

### Outcomes

A case report form was designed to collect sociodemographic and clinical characteristics from the sites’ health records, and this information was confirmed during a clinical interview. HDRS-17 scores (range: 0-52, with the highest score indicating severe depression) obtained at baseline and each visit (at weeks 2, 4, 8, 12, and 24) were used to inform 2 primary outcomes measures: estimated time from randomization to response (defined as ≥50% reduction in HDRS-17 scores from baseline) and estimated time to remission (defined as a HDRS-17 score of ≤7).^15^ Secondary outcomes included adherence at each treatment visit (based on pill counts), rates of response and remission, changes in the severity of depressive symptoms (ie, change in HDRS-17 score), and time to discontinuation from the study from all causes. In addition, using a checklist, 6 common adverse effects (dry mouth, diarrhea or constipation, dizziness or drowsiness, loss of appetite or nausea, headache, and excessive sweating) were assessed at baseline and each treatment visit.

At each study site, all outcome measures were collected by 2 experienced assessors who were blind to the treatment group. The MBC coordinator at each site reminded participants at each visit about nondisclosure of their intervention group to the assessor.

### Statistical Analysis

Full intention-to-treat analyses were conducted. Time-to-event outcomes (response, remission, and all-cause discontinuation) were analyzed using Kaplan-Meier survival curves. Between-group differences were assessed using the log-rank test and Cox proportional hazards regression model. The Cox model was adjusted for age, marital status, and baseline HDRS-17 scores to account for potential confounding. Rates of adherence, response, remission, and adverse effects were compared with χ^2^ tests. Baseline HDRS-17 scores, antidepressant doses, number of clinic visits, changes in HDRS-17 at week 24, and number of adverse effects were compared with independent, 2-tailed *t* tests or Mann-Whitney tests, as appropriate. The significance threshold was 2-tailed *P* < .05 for all analyses. All analyses were conducted using SPSS for Windows, version 20.0 (IBM Corp).

## Results

Between September 2022 and January 2024, 351 patients were screened and 166 met eligibility criteria. Of those eligible, 154 were randomly assigned to MBC (n = 76) or standard care (n = 78) (Figure 1). The 2 groups appeared similar in baseline sociodemographic and clinical characteristics (Table 1). Overall, patients included 105 females (68.2%) and 49 males (31.8%), with a mean (SD) age of 34.5 (10.5) years.

**Table 1.** Baseline Demographic and Clinical Characteristics of Participants Receiving Measurement-Based Care or Standard Care for Major Depressive Disorder

Characteristic	Participants, No. (%)		
	Overall sample (N = 154)	MBC group (n = 76)	Standard care group (n = 78)
Sex			
Female	105 (68.2)	50 (65.8)	55 (70.5)
Male	49 (31.8)	26 (34.2)	23 (29.5)
Family system: nuclear	95 (61.7)	46 (60.5)	49 (62.8)
Marital status: married	108 (70.1)	56 (73.7)	52 (66.7)
Socioeconomic status: lower income	86 (55.8)	46 (60.5)	40 (51.3)
Initial antidepressant			
Paroxetine	104 (67.5)	42 (55.3)	62 (79.5)
Mirtazapine	50 (32.5)	34 (44.7)	16 (20.5)
Age, mean (SD), y	34.5 (10.5)	35.7 (11.2)	33.2 (9.6)
No. of years of education, mean (SD)	7.0 (5.0)	7.0 (6.0)	7.0 (5.0)
Family members in household, mean (SD)	7.0 (4.0)	7.0 (4.0)	7.0 (4.0)
Annual participant income, median (IQR), US $	1127.48 (901.98-1409.35)	1127.48 (969.63-1634.85)	901.98 (721.59-1352.98)
Annual household income, median (IQR), US $	1352.98 (1127.48-2254.96)	1352.98 (1127.48-2254.96)	1352.98 (1127.48-2254.96)
Time since MDD diagnosis, median (IQR), wk	52.00 (13.75-149.50)	52.00 (24.75-162.50)	52.00 (12.25-104.00)

Abbreviations: MBC, measurement-based care; MDD, major depressive disorder.

Mean (SD) antidepressant doses (in amitriptyline equivalents) were significantly higher in the MBC group than in the standard care group at weeks 8 (153.9 [134.0] mg/d vs 112.7 [52.4] mg/d; *P* = .02), 12 (153.9 [133.1] mg/d vs 113.6 [52.3] mg/d; *P* = .03), and 24 (152.3 [134.1] mg/d vs 113.8 [52.6] mg/d; *P* = .04) (Table 2). Treatment adherence, as measured by percentages of pills consumed based on pill counts, was high and similar at all points except at weeks 6 and 12 (eTable 2 in Supplement 2). The number of clinic visits was significantly higher in the MBC group from week 5 to week 12, with a median (IQR) of 2 (2-2) visits in the MBC group and 1 (1-2) visit in the standard care group (Table 2).

**Table 2.** Antidepressant Doses and Hamilton Depression Rating Scale Scores for Participants Receiving Measurement-Based Care or Standard Care for Major Depressive Disorder

Outcome	Mean (SD)		*P* value
	MBC group (n = 76)	Standard care group (n = 78)	
**Baseline**			
HDRS-17 score	24.1 (5.0)	23.4 (4.3)	NA
**Wk 2**			
No.	68	72	NA
Dose, mg/d	96.3 (57.5)	98.3 (62.6)	.85
HDRS-17 score	12.2 (5.3)	14.6 (8.9)	.05
Visits from baseline to wk 2, median (IQR)	1 (1-1)	1 (1-1)	.99
**Wk 4**			
No.	66	71	NA
Dose, mg/d	130.3 (112.9)	112.3 (53.8)	.24
HDRS-17 score	8.3 (4.9)	12.6 (8.1)	<.001
Visits from wk 3 to wk 4, median (IQR)	1 (1-1)	1 (1-1)	.99
**Wk 8**			
No.	64	68	NA
Dose, mg/d	153.9 (134.0)	112.7 (52.4)	.02
HDRS-17 score	7.7 (5.2)	10.4 (7.4)	.02
Visits from wk 5 to wk 8, median (IQR)	2 (2-2)	1 (1-2)	<.001
**Wk 12**			
No.	64	68	NA
Dose, mg/d	153.9 (133.1)	113.6 (52.3)	.03
HDRS-17 score	6.9 (5.3)	9.2 (6.9)	.04
Visits from wk 9 to wk 12, median (IQR)	2 (2-2)	1 (1-2)	<.001
**Wk 24**			
No.	64	67	NA
Dose, mean (SD), mg/d	152.3 (134.1)	113.8 (52.6)	.04
HDRS-17 score, mean (SD)	5.6 (4.2)	6.7 (4.7)	.14
Visits from wk 13 to wk 24, median (IQR)	1 (1-1)	1 (1-1)	.99

Abbreviations: HDRS-17, 17-item Hamilton Depression Rating Scale; MBC, measurement-based care; NA, not applicable.

**Figure 2.**
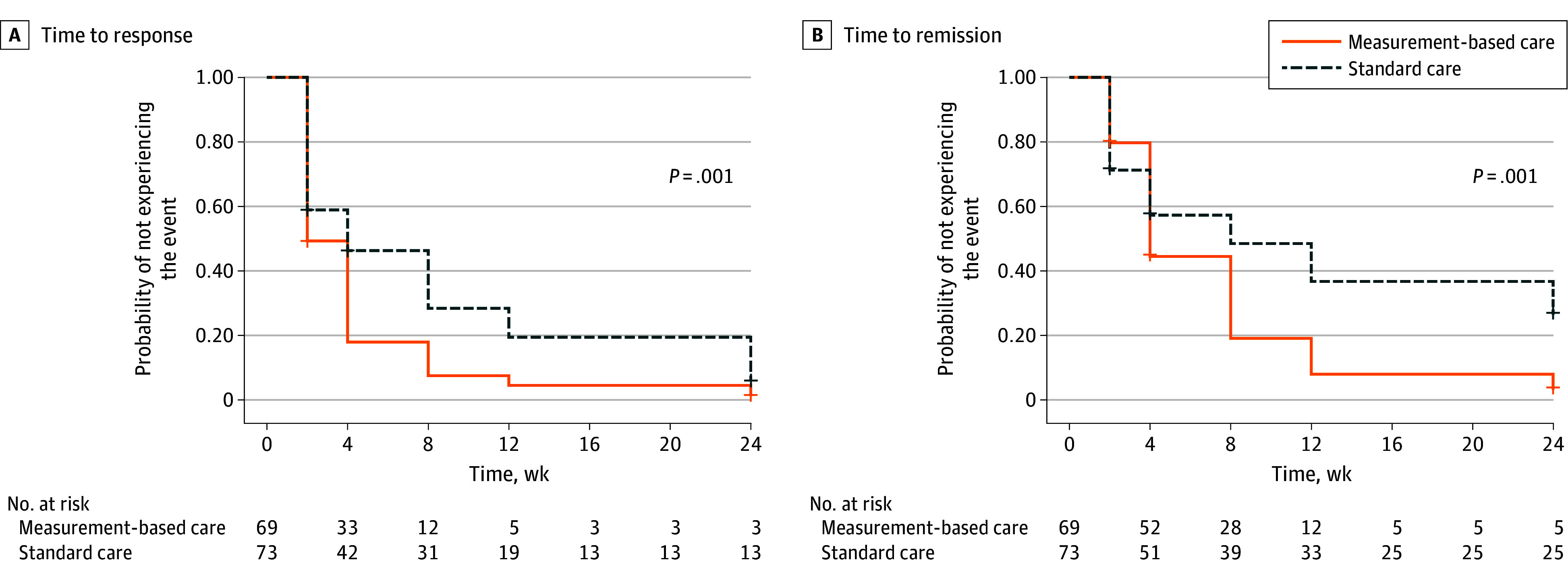
Kaplan-Meier Survival Curve for Time to Response and Time to Remission by Treatment Group

The median (IQR) time to response was 2 (2-4) weeks in the MBC group and 4 (2-12) weeks in the standard care group. The median (IQR) time to remission was 4 (4-8) weeks in the MBC group and 8 weeks (2 weeks to no remission) in the standard care group. The estimated time intervals for response and remission in the 2 groups in the Kaplan-Meier analysis are illustrated in Figure 2. The difference between the survival curves was significant for both time to response (log rank test, χ^2^_1_ = 10.21; *P* = .001) and time to remission (χ^2^_1_ = 11.08; *P* = .001). In the Cox proportional hazards regression model, the likelihood of response (hazard ratio [HR], 1.53; 95% CI, 1.06-2.20; *P* = .02) and remission (HR, 1.80; 95% CI, 1.21-2.68; *P* = .004) were significantly higher in the MBC group than in the standard care group.

After week 24, the response rates were 89.1% in the MBC group and 86.6% in the standard care group (χ^2^_1_ = 0.19; *P* = .79), for an overall response rate of 87.8%. The remission rates were 73.4% in the MBC group and 62.7% in the standard care group (χ^2^_1_ = 1.74; *P* = .20), for an overall remission rate of 67.9%. After week 24, the reduction in mean HDRS-17 scores was larger in the MBC group than in the standard care group (−18.1 [95% CI, 16.4-19.6] points vs −17.0 [95% CI, 15.6-18.5] points; *t*_129_ = 0.71; *P* < .001) (Table 3)

**Table 3.** Efficacy Outcomes for Participants Receiving Measurement-Based Care or Standard Care for Major Depressive Disorder

Measure	Participants, No. (%)		*P* value
	MBC group	Standard care group	
Response			
Wk 12	58 (90.6)	46 (67.6)	<.001
Wk 24	57 (89.1)	58 (86.6)	.79
Remission			
Wk 12	46 (71.9)	35 (51.5)	.02
Wk 24	47 (73.4)	42 (62.7)	.20
Estimated time to response, median (IQR), wk	2 (2-4)	4 (2-12)	.001^a^
Estimated time to remission, median (IQR), wk	4 (4-8)	8 (2-NR)^b^	.001^a^
Change in HDRS-17 score, mean (95% CI), points	18.1 (16.4-19.6)	17.0 (15.6-18.5)	<.001

Abbreviations: HDRS-17, 17-item Hamilton Depression Rating Scale; MBC, measurement-based care; NR, no remission.

^a^ Difference between survival curve of Kaplan-Meier analysis.

^b^ No remission within the follow-up period of 24 weeks.

In the Kaplan-Meier analysis of all-cause discontinuation, the difference between the survival curves in both groups was not significant (χ^2^_1_ = 0.10; *P* = .70) (eFigure in Supplement 2). The number and frequency of any type of adverse events were similar across both groups (eTable 3 in Supplement 2).

## Discussion

This RCT compared the outcomes of MBC and standard care for the pharmacotherapy of MDD in Pakistan. The results support that MBC, including a sequential treatment protocol with MBC coordinators facilitating physicians’ adherence to this protocol, can be successfully integrated into clinical practice and improve patient outcomes in an LMIC, such as Pakistan. Time to response and time to remission were significantly shorter in the MBC group than in the standard care group. These results confirm and extend the results of a previous trial conducted by Guo et al^8^ in China. They are clinically meaningful, as they suggest that MBC reduces the duration of depressive symptoms in an LMIC.

There were a higher number of clinic visits and higher antidepressant doses in the MBC group, without a higher frequency of treatment discontinuation or adverse effects. Similar to the Guo et al^8^ trial, the higher doses in the first 4 weeks of treatment may have led to faster symptom reduction in the MBC group. Inadequate antidepressant doses have been shown to contribute to poor outcomes in a subgroup of patients with MDD.^16^ The significantly higher rates of response and remission in the MBC group at week 12 support an earlier assertive approach to treatment of MDD. The similar rates of treatment discontinuation and adverse effects in the 2 treatment groups also indicate that a higher burden of adverse effects did not compromise the more assertive antidepressant treatment in the MBC group.

The results of both our primary outcomes (ie, time to response and time to remission) and early secondary outcomes (ie, rate of response and rate of remission at week 12) are congruent with the findings of the Guo et al^8^ trial, with almost identical rates of remission in the MBC group at week 12 (73.4% in the present trial vs 73.8% in Guo et al^8^). By contrast, in the current trial, rates of response and remission were no longer significantly different at week 24, when the rate of remission in the standard care group had increased to 62.7% (vs 28.8% in the Guo et al^8^ trial). Differences in study setting and populations may explain this discrepancy. The Guo et al^8^ trial was conducted in a large urban university–affiliated teaching hospital in Beijing, China, whereas the present trial was conducted across several Pakistani cities, in clinics and Basic Health Units that serve large suburban and rural populations. Previous studies have shown the therapeutic effects of attentive care through repeated study visits in clinical trials,^17,18,19^ with each additional visit contributing to further symptom improvement.^20^ While participants in the control group did not complete repeated clinical measures of their depressive symptoms or adverse effects, they completed repeated research assessments of their symptoms, which may also have contributed to their high response and remission rates.^21^ These nonspecific therapeutic effects may be particularly relevant in low-resource settings, such as Pakistan, where more than 90% of individuals (particularly those in suburban or rural areas) do not have access to evidence-based mental health care.^22^

In addition to repeated monitoring of their clinical symptoms and adverse effects, participants in the MBC group may have benefited from the MBC treatment protocol and MBC coordinators who facilitated physician adherence to this protocol. The timely feedback the coordinators provided to physicians treating participants in the MBC group likely helped to overcome common barriers, such as clinical inertia. This benefit highlights that successful deployment of MBC may require not only structured clinical measurements but also dedicated personnel to facilitate implementation of treatment protocols.^23^

### Strengths and Limitations

Our decision to replicate a previous RCT of MBC was driven in part by the need to address what has been called the replicability crisis in mental health research.^24,25,26^ This focus allowed us to enroll a relatively modest sample size. The high retention rate (85% overall), pragmatic design, and implementation in a low-resource setting are additional strengths of this trial and enhance the generalizability of our findings. Most RCTs of pharmacotherapies, including antidepressants, are conducted in HICs, limiting their international representativeness.^27^

Limitations of this trial are linked to its strengths. We adopted the study design of the Guo et al^8^ trial in China and used the same 2 antidepressants (paroxetine and mirtazapine), which are not the antidepressants most commonly used in Pakistan or other countries.^28^ This replication may limit generalizability to clinical prescribing practices. We also used the same sample size that was based on expected rates of remission (ie, a secondary outcome) rather than the primary outcomes of time to response and time to remission. These outcomes were focused on depressive symptoms rather than broader measures of function or quality of life. Another limitation of the trial is the absence of a formal assessment of interrater reliability. To mitigate this gap, all blinded assessors underwent centralized training and certification prior to the study to ensure consistency in administering and scoring the HDRS-17. Regular supervision and calibration meetings were also held to minimize variability across raters. Finally, early in the trial, participants in the MBC group experienced high rates of response and remission that can be explained by the data-driven clinical decision-making and the monitoring of physician adherence to the treatment protocol in this group. Later in the trial, similar high rates of response and remission were also observed in the standard care group, possibly due to the structured nature of RCTs, including frequent follow-up visits and research assessments of symptoms. Because of these enhancements to standard care, outcomes in the standard care group may not reflect the outcomes obtained under actual clinical conditions.^21^

## Conclusions

In this trial of adults with MDD in Pakistan, MBC accelerated the time to response and time to remission compared with standard care. Given its scalable nature, MBC offers a viable solution to the need for effective mental health interventions in LMICs, where resources are often constrained and populations face substantial barriers to accessing mental health care. Future studies need to confirm the clinical effectiveness of MBC and assess its cost-effectiveness across various LMICs.

## Notes

Supplement 1.
Trial Protocol and Statistical Analysis Plan

Supplement 2. **eTable 1**. The Measurement-Based Care Schedule **eTable 2.** Medication Adherence as Assessed via Percentage of Pills Consumed at Each Time Point **eTable 3.** Adverse Effects Among Participants Receiving Measurement-Based Care or Standard Care **eFigure.** Estimated Median Time to Discontinuation by Kaplan-Meier Analysis

Supplement 3.
Data Sharing Statement
