# Errors in Table 1

**DOI:** 10.1001/jamanetworkopen.2026.26785

**Published:** 2026-07-14

In the Original Investigation titled “Measurement-Based Care to Enhance Antidepressant Treatment Outcomes in Major Depressive Disorder: A Randomized Clinical Trial”^1^ published on September 2, 2025, the values for annual participant and household income and time since diagnosis (median and IQR) reported in Table 1 were calculated using the SPSS “HAVERAGE” (weighted average/interpolated) method, but should have been calculated using simple non-interpolated methods. The values have been recalculated accordingly and the article has been corrected.^1^
