# Supplementary material for: Measurement-Based Care to Enhance Antidepressant Treatment Outcomes in Major Depressive Disorder: A Randomized Clinical Trial
Source: JAMA Netw Open. 2025 Sep 2;8(9):e2529427. doi: 10.1001/jamanetworkopen.2025.29427 (PMC12406064; doi:10.1001/jamanetworkopen.2025.29427)
Supplement: Supplement 1. — Trial Protocol and Statistical Analysis Plan [file jamanetwopen-e2529427-s001.pdf]

Measurement-based care vs. standard care for major depressive disorder in Pakistan: protocol for a randomized control trial

M. Ishrat Husain[1,2], Madeha Umer[2,3], Ameer B. Khoso[3], Tayyeba Kiran[3], M. Omair Husain[1,2], Benoit H. Mulsant[1,2], Nusrat Husain[4,5], Nasim Chaudhry[3] Imran B. Chaudhry[3,4,6]

1. *Campbell Family Mental Health Research Institute, Centre for Addiction and Mental Health, Toronto, Ontario, Canada*
2. *Department of Psychiatry, University of Toronto, Toronto, Ontario, Canada*
3. *Pakistan Institute of Living and Learning, Karachi, Pakistan*
4. *Division of Psychology and Mental Health, School of Health Sciences, University of Manchester, Manchester, UK*
5. *Mersey Care NHS Foundation Trust,*
6. *Department of Psychiatry, Ziauddin University, Karachi, Pakistan*

Corresponding author:
Madeha Umer
Pakistan Institute of Living and Learning, Karachi, Pakistan
madeha.umer@pill.org.pk

Abstract

Background: Low and middle-income countries (LMICs) hold the majority of disease burden attributed to major depressive disorder (MDD). Despite this, there remains a substantial gap for access to evidence-based treatments for MDD in LMICs like Pakistan. Measurement-based care (MBC) incorporates systematic administration of validated outcome measures to guide treatment decision making and is considered a low-cost approach to optimise better clinical outcomes for individuals with MDD but there is a paucity of evidence on the efficacy of MBC in LMICs.

Methods: This protocol highlights a randomized trial which will include Pakistani outpatients with moderate to severe major depression. Participants will be randomised to either MBC (guided by schedule), or standard treatment (guided by clinicians' judgement), and will be prescribed with paroxetine (10–60mg/day) or mirtazapine (7.5–45mg/day) for 24 weeks. Outcomes will be evaluated by raters blind to study protocol and treatment.

Discussion: With increasing evidence from high-income settings supporting the effectiveness of MBC for MDD, it is now necessary to explore its feasibility, utility. and efficacy in low-resource settings. The results of the proposed trial could inform the development of a low-cost and scalable approach to efficiently optimise outcomes for individuals with MDD in Pakistan.

Keywords: Measurement-based care, Treatment guidelines, Depression, Major depressive disorder, Antidepressant treatment

Trial registration: NCT05431374

Introduction

Major depressive disorder (MDD), a leading cause of disability worldwide, is indicated as one of the two most disabling mental disorders by the Global Burden of Diseases, Injuries, and Risk Factors Study (GBD) 2019 (Vos et al., 2020). The GBD study further highlights an increase in incidents of depression worldwide, from 172 million in 1990 to 25.8 million in 2017, representing a drastic increase of 49.86% (Liu et al., 2020). This sharp increment, coupled with the debilitating physical and mental symptoms of MDD, translates into harrowing global health burden. These high rates of prevalence fare worse for low-income and densely populated countries like Pakistan, with a population of more than 220 million. Various epidemiological studies from Pakistan have reported a prevalence of 20-50% for depression (Ahmed et al., 2016; Gadit & Mugford, 2007; Karmaliani et al., 2009). This prevalence occurs in the context of limited access to mental health care with less than 500 psychiatrists for the entire Pakistani population (Sikander, 2020). The paucity of mental health professionals in Pakistan creates a massive treatment gap, which can only be minimized by standardized and cost-effective interventions that result in higher rates of response and remission.

However, despite several effective pharmacological and psychosocial interventions available globally, only about one-third of depressed patients achieve remission (Xiao et al., 2021). Clinical evidence indicates low remission and high dropout rates due to inadequate dose and/or duration of antidepressant medication treatment (R. C. Kessler et al., 2003; Ormel et al., 2019). Quick changeovers from one antidepressant to another or an unnecessarily prolonged treatment trials result in a practice bias (Rush et al., 2006; Trivedi, Fava, et al., 2006), therefore clinical trials exploring the efficacy of depression care specialists report only a modest remission rate of 15% to 35% (Katon et al., 2010; Trivedi et al., 2004). Subjective outcome assessments also lead to wide variances in symptomology, functionality, side-effect frequency and burden (Biggs et al., 2000). This calls for a need to establish scalable clinical management practices which utilize biopsychosocial assessments, formulate a differential diagnosis, and provide evidence-based treatments for patients with MDD (Hong et al., 2021).

Major treatment guidelines from high-income countries emphasize the importance of 'Measurement-Based Care' (MBC), which utilizes outcomes based on validated clinical instruments to

guide treatment decisions (Aboraya et al., 2018; Gelenberg, 2010; Kennedy et al., 2016). This concept was derived from the stepwise treatment algorithm developed through the Sequenced Treatment Alternatives to Relieve Depression (STAR*D) trial(Trivedi, 2009; Trivedi, Rush, et al., 2006; Trivedi & Daly, 2007). The large-scale trial funded by the US National Institute of Mental Health examined treatment options in an algorithmic manner with the aim to guide the next steps for treatment of patients with MDD who have an inadequate response to an antidepressant. The trial reported significantly improved outcomes, better-informed treatment decisions and greater remission rates in patients with chronic depression. Similarly, in a study of 120 patients with moderate to severe MDD in China, significantly more patients receiving MBC achieved response (86.9% compared with 62.7%) and remission (73.8% compared with 28.8%) compared to those receiving standard care(Guo et al., 2015). Time to response and remission were significantly shorter with MBC (for response, 5.6 weeks compared with 11.6 weeks, and for remission, 10.2 weeks compared with 19.2 weeks). There was also a significantly larger decrease in Hamilton Depression Rating Scale (HDRS) scores (- 17.8 compared to -13.6) in the MBC group. The MBC group had significantly more treatment adjustments (44 compared with 23) and higher antidepressant dosages from week 2 to week 24 (Guo et al., 2015). MBC has also been found to help patients gain insight into their illness course; those who completed self-reports of depressive symptoms expressed that the measures allowed them to quantify their symptoms and gain a better understanding of their experience with depression (Dowrick et al., 2009). The better educated patients are about these measurements, the more they adhere to a treatment plan (Trivedi et al., 2020). MBC may also be a valuable tool for facilitating collaborative care among providers within and across organizations (Katon et al., 2010). Given MBC's potential to improve outcomes, it may be one of the minimal interventions necessary for maximal improvement in clinical outcomes in patients with MDD (R. Kessler & Glasgow, 2011).

While significant evidence for effectiveness of MBC is found in clinical settings from high and middle-income countries, assessments of MBC compared with usual care for the treatment of MDD are yet to be completed in low-resource settings like LMICs. Given the high prevalence of MDD, economic burden and limited resources, countries like Pakistan can benefit immensely from assessment and implementation of MBC to enhance outcomes for patients.

Methods

The aim of this trial is to determine the efficacy and safety of MBC in patients with MDD in comparison with standard care in Pakistan. In order to reduce the variance found in treatment-as-usual and isolate the impact of MBC, standard care for this trial will limit medication choices to either paroxetine or mirtazapine (as per previously published trials). We hypothesize that, compared to standard care group, the MBC group will have significantly higher rates and shorter times to response and remission, along with lower rates of dropout and side effect burden.

Study design and setting

This will be a multi-center, with assessors blind to protocol and treatment group, parallel arm, randomized controlled trial (RCT). The study is a direct replication of a study conducted by Guo et al. (2015) in China (Guo et al., 2015).

Participants

Participants will be recruited from psychiatric units of teaching and non-teaching hospitals and primary care settings such as General Physician (GP) clinics and Basic Health Units (BHUs) in 6 centers: Karachi (population 23 million), Lahore (population 10 million), Rawalpindi (population 3 million), Hyderabad (population 2 million) and Quetta (population 1 million) and Multan (1.8 million). By recruiting participants from across the country, we are confident that the sample will be representative of Pakistani patients with MDD.

Inclusion Criteria

Adult outpatients (18-65 years of age), diagnosed with nonpsychotic MDD (established by treating psychiatrists and confirmed by a checklist based on DSM-5 criteria at screening) and with a score of at least 18 on the 17-item Hamilton Depression Rating (HDRS-17) (administered as part of the routine pre assessment questionnaire) will be the inclusion criterion of this study. These individuals should also be able to communicate effectively and give written informed consent and be a resident of the trial catchment area.

Exclusion criteria

We will exclude individuals who have; lifetime history of drug or alcohol dependence; diagnosis of bipolar, psychotic, obsessive-compulsive, or eating disorders confirmed with DSM-5 criteria, history of a lack of response or intolerance to either of the two protocol antidepressants (paroxetine and mirtazapine), suicide attempts in the current depressive episode, any major medical condition contraindicating the use of the protocol antidepressants, or are currently pregnant or breastfeeding.

Sample size

The sample size of 120 participants for this exploratory trial is based on the study conducted by Guo et al. 2015(Guo et al., 2015).

Randomization and masking

Participants will be randomized in a 1:1 allocation to measurement-based vs. standard-care arm. Randomization will be computer generated and use a random permuted block method with variable block sizes. Given the potential differences in recurrence rates in these groups, the sample will be stratified by site and self-reported sex. We will assess other potential prognostic characteristics (age, duration of illness, number of previous episodes, gender) in our a priori subgroup analyses.

Given the nature of the intervention, it will be impossible to blind clinicians in participating centers or the participants, however this will be an assessor blind trial. The assessors will be trained to use instruments, and will carry out assessments at an arms-length from treating physicians. Before assessments, participants will be asked not to reveal any information about treatment to assessors. In case of unintentional unblinding, we will assign new outcome assessors. Statistical analysis will be partially blind (knowing treatment groups, but not what each group is).

Study Intervention and Procedure

After providing informed consent, eligible patients will enter a one-week washout phase to eliminate any effects of psychotropic medications previously taken. After washout week, the patients will be assigned to treatment arms based on randomization schedule, where they will receive either open-label paroxetine

(10–50mg/day) or open-label mirtazapine (7.5–45mg/day). All patients will be followed for 24-weeks after randomization. This dosage range is recommended by the US Food and Drug Authority (*Paxil - Highlights of Prescribing Medication*, 2022; *Remeron -Highlights of Prescribing Medication*, 2022). Paroxetine is a commonly prescribed antidepressant, working as a selective serotonin reuptake inhibitor, while Mirtazapine, which works as an alpha-2 antagonist, has a different mechanism of action. Similar to the Guo et. al study (Guo et al., 2015), the decision about the prescription of drug and dosage will be made by the treating psychiatrists/GP. Depending on the duration of prescribed dosage, symptoms and side effects, the psychiatrist/GP will make adjustments. In case of intolerability or inefficacy, one medication change between paroxetine and mirtazapine will be allowed. Short-acting benzodiazepines, prescribed occasionally for agitation, anxiety, and insomnia, will be permitted, as well as any other medications not affecting the CNS.

Measurement-based Care

At every outpatient visit, patient's feedback will be recorded through the 16-item Quick Inventory of Depressive Symptomatology–Self-Report (QIDS-SR) (Rush et al., 2003) and the Frequency, Intensity, and Burden of Side Effects Rating scale (FIBSER) (Wisniewski et al., 2006). The QIDS-SR is used to measure depressive symptoms in the past one week, with higher scores indicating higher severity (Rush et al., 2003). FIBSER is completed by the patient to report antidepressant side effects on the domains of frequency, intensity, and burden(Wisniewski et al., 2006). A total score >4 is indicative of current treatment being unsuitable and requires revision to dosage/medication (Trivedi, 2009).

The patient's responses on these self-report measures will inform and guide physician's decisions. Following a stepwise approach, the treatment schedule will outline the dosage adjustment, and medication changes needed to mitigate the side effects, and promote therapeutic benefit for each patient. Originally driven from the STAR*D project (www.star-d.org), the treatment schedule used by the Guo et. al. (2015) study (Guo et al., 2015) will facilitate the treatment schedule (Table 1), for this study. However, the starting dosage has been modified following a discussion with the stakeholders, which highlighted patient preference for slower dose titration when initiating preference. Treating physicians will undergo a two-day training to get acquainted with use of MBC schedule. Moreover, an independent research coordinator will be designated at each site to compare the physician's clinical notes against the MBC treatment guidelines to ensure compliance and standardization. In case of any deviation from the MBC schedule, the physician will be notified and asked to make necessary treatment amendments.

Table 1. The Measurement-Based Care Schedule

Time Point and Measure	Clinical Status	Treatment Plan	
		Paroxetine	Mirtazapine
**Week 0**		Start at 10 mg- 20 mg/day	Start at 7.5 mg- 15mg/day, increase to 15 mg/day by week 1
**Week 2**			
Outpatient visit,		Increase or maintain dose at 20 mg/day	Increase or maintain dose at 15 mg/day
**Week 4**			
QIDS-SR score, ≤5	Remission	Continue current dose	Continue current dose
QIDS-SR score, 6–8	Partial response	Continue current dose or consider increasing to 30 mg/day	Continue current dose or consider increasing to 45 mg/day
	SEs intolerable	Continue current dose and address SEs, or switch to mirtazapine	Continue current dose and address SEs, or switch to paroxetine
QIDS-SR score, ≥9	Nonresponse	Increase to 30 mg/day or switch to mirtazapine	Increase to 45 mg/day or switch to paroxetine
	SEs intolerable	Switch to mirtazapine	Switch to paroxetine
**Week 6**			
QIDS-SR score, ≤5	Remission	Continue current dose	Continue current dose
QIDS-SR score, 6–8	Partial response	Continue current dose or consider increasing to 40 mg/day	Continue current dose or consider increasing to 45 mg/day
	SEs intolerable	Continue current dose and address SEs, or switch to mirtazapine	Continue current dose and address SEs, or switch to paroxetine
QIDS-SR score, ≥9	Nonresponse	Increase to 40 mg/day	Increase to 45 mg/day or switch to paroxetine
	SEs intolerable	Switch to mirtazapine	Switch to paroxetine
**Week 8**			
QIDS-SR score, ≤5	Remission	Continue current dose	Continue current dose
QIDS-SR score, 6–8	Partial response	Continue current dose or consider increasing to 50 mg/day	Continue current dose or consider increasing to 45 mg/day
	SEs intolerable	Switch to mirtazapine	Switch to paroxetine
QIDS-SR score, ≥9	Nonresponse	Switch to mirtazapine	Switch to paroxetine
	SEs intolerable	Switch to mirtazapine	Switch to paroxetine
**Week 10**			
QIDS-SR score, ≤5	Remission	Continue current dose	Continue current dose
QIDS-SR score, 6–8	Partial response	Continue current dose or consider increasing to 60 mg/day	Continue current dose or consider switch to paroxetine
	SEs intolerable	Switch to mirtazapine	Switch to paroxetine
QIDS-SR score, ≥9	Nonresponse	Switch to mirtazapine	Switch to paroxetine
	SEs intolerable	Switch to mirtazapine	Switch to paroxetine
**Week 12**			
QIDS-SR score, ≤5	Remission	Continue current dose and follow up	Continue current dose and follow up
QIDS-SR score, 6–8	Partial response	Continue current dose and follow up, or consider switch to mirtazapine	Continue current dose and follow up, or consider switch to paroxetine
	SEs intolerable	Switch to mirtazapine	Switch to paroxetine

Time Point and Measure	Clinical Status	Treatment Plan	
		Paroxetine	Mirtazapine
QIDS-SR score, ≥9	Nonresponse	Switch to mirtazapine	Switch to paroxetine
	SEs intolerable	Switch to mirtazapine	Switch to paroxetine

Control/Standard-care

Local medical, psychiatric and family medicine services provide routine care according to their clinical judgment and available resources. Standard-care will be ascertained by the participant's treating physician based on the patient's clinical needs. Research staff will record the nature and intensity of standard-care delivered to each participant. In current practice, MDD patients are not routinely referred for any psychological therapies in Pakistan. Standard-care in Pakistan largely comprises of pharmacotherapy.

Outcome measures

A form will be designed to collect sociodemographic and clinical characteristics from the medical records, and this information will be confirmed in a clinical interview. HDRS scores will be used to inform two primary outcomes measures: estimated time from randomization to response (≥ 50% reduction from baseline) and remission (HDRS score ≤ 7) (Hamilton, 1960).

Secondary outcome measures will include: severity of depressive symptoms (HDRS score), severity of manic or hypomanic symptoms according to the (YMRS score) (Young et al., 1978). An additional checklist with six common side effects (dry mouth, diarrhea or constipation, dizziness or drowsiness, loss of appetite or nausea, headache, and excessive sweating), along with FISBER, will be used to measure side effects at each treatment visit. The pill count method will be used to measure treatment adherence.

Assessment Procedure

This trial will include two experienced treatment-blind assessors at each site, who will independently administer the mentioned outcome measures at baseline and at 2, 4, 8, 12, and 24 weeks. The research coordinator at each site will advise and remind patients at each visit about non-disclosure of their treatment groups to the assessor.

Statistical analysis

We will analyze the data using SPSS for Windows, version 20.0 (IBMCorp., Armonk, N.Y.). Similar to the Guo et al. (2015) trial, we will use independent-sample t-tests, Mann-Whitney U tests, and chi-square tests, to compare sociodemographic and clinical characteristics, discontinuation, response and remission rates, and side effects as appropriate. Full intent-to-treat analyses will be carried out to determine all-cause and specific-cause discontinuation. Estimated time from randomization to response and remission will be calculated using Kaplan-Meier survival analyses, and a comparative analysis will be facilitated using the Cox proportional hazards regression model. Analysis of covariance will be used to compare the changes in HDRS and YMRS scores between the two groups. Additional sub-group analysis might be carried out to understand group-differences due to age, gender, duration of illness, and number of previous episodes. The significance threshold will be $p<0.05$ (two-tailed) for all analyses. A treatment-blind statistician will carry out interim quality checks and efficacy analysis, while final evaluation will be carried out after completion of followups for all recruited participants.

Ethical and safety considerations

The trial will be conducted and reported as per recommendation of the CONSORT statement for RCTs. National Bioethics Committee (NBC) of Pakistan has given full ethics approval. All members of the research team will comply with the International Conference on Harmonization Good Clinical Practice (ICH-GCP) guidelines. Research staff will be trained on Good Clinical Practices (GCP) and will not begin data collection until the GCP certification is successfully completed. All the information provided by the participants will be kept confidential and authorization will be required prior to access. Patient identifying information will not be published. Participation to the trial will be voluntary and participants will have the right to withdraw from the study at any time, without giving any reason. Withdrawal from the trial will have no effect in routine care. Informed consent forms will be in the local language for those participants who can read. Participants, who are unable to read and write, will be provided with verbal information (in the local language) that will encompass all aspects of the participant written informed consent form. The participants will then sign the consent form and a caregiver will sign alongside. However, if they cannot write, the participant's caregiver will sign alongside the participant's thumbprint. The PIS and the participant informed consent form will include details of the purpose of the study; the opportunity to ask questions; voluntary participation in the study and the right to withdraw from the study at any time, for any reason; and privacy and confidentiality. All participants can decide to participate right

away if they want to, or they will be given at least 48 hours to read and discuss queries with family or the research team before a decision for participation is made.

We do not anticipate any major risks related to the intervention. However, we acknowledge that potentially vulnerable people with mental illness will be participating in the trial. Some topics discussed or questions asked in the sessions/assessments may cause distress, and in such instances, appropriate support will be provided. We will map available mental health resources ensuring pathways to care. The research team has considerable clinical and research experience, including the management of difficult situations arising in research interventions and interviews. In case participant recruited to this study experience active suicidal ideation or subsequently identified as needing more intensive treatment will be appropriately referred for psychiatric evaluation and treatment. Patients will be removed from the study if they report suicidal attempt, became pregnant, developed a severe medical condition, or suffered from newly emerging side effects that they find intolerable and that could not be managed. Patients who will be removed from the study will receive antidepressant treatment as appropriate as part of clinical care. Research staff will be provided with regular supervision throughout the duration of the project. We already have study protocols in place that include details about how to manage difficult situations arising in research, safety and lone working arrangements. In the event of any study-related injury or adverse event, intervention and treatment will be available from medical staff. Any adverse events will be reported to the REB within the required timeframe.

Data management and monitoring

Each participant will be assigned a unique study identification (ID) number and identifying information will be placed in locked cupboards separate to other study data that will only be accessible to the authorized researchers. Paper copies of assessment tools will all be stored in locked filing cabinets in PILL premises. All anonymized data will be stored in encrypted and password protected computers. We will convene an independently chaired trial steering committee (TSC) to approve and provide oversight of the trial throughout its various stages. The TSC will include the PI, patient representative, Co-Is, an independent statistician, and an independent chair. The Data Safety and Monitoring Committee (DSMC) will monitor data and advise the TSC on

whether there are any ethical or safety reasons for why the trial should not continue. Both committees will meet twice in the first year and then once a year thereafter.

Discussion

Empirical evidence from several trials lends support to patients gaining favorable outcomes when assigned to MBC compared to standard-care treatments (Zhu et al., 2021). This stepwise treatment, not only helps clinicians understand the patterns which lead to improvement, but also accelerates the process of sifting through various regimens to reach an effective treatment plan for the patient (Zhu et al., 2021). This is especially important for patients from low resource settings, who are already walking on the tightrope of low access and inconsistent resources in mental health. Hence, this trial aims to inform the feasibility and efficacy of implementing MBC in everyday clinical practice for patients with MDD in Pakistan.

Declarations

Abbreviations

MDD: Major Depressive Disorder; GBD: Global Burden of Diseases, Injuries, and Risk Factors Study; MBC: Measurement-based Care; STAR*D: Sequenced Treatment Alternatives to Relieve Depression; SCID 5: Structured Clinical Interview for DSM-5; QIDS-RS: Quick Inventory of Depressive Symptomatology–Self-Report (QIDS-SR); (FIBSER): Frequency, Intensity, and Burden of Side Effects Rating scale; HDRS: Hamilton Depression Rating Scale; YMRS: Young Mania Rating Scale; TSC: Trial Steering Committee; DSMC: Data Safety and Monitoring Committee: (NBC) National Bioethics Committee ICH-GCP)International Conference on Harmonization Good Clinical Practice; GCP: Good Clinical Practices

Acknowledgements

Not applicable.

Authors' contributions

MI conceptualized, managed, and coordinated the study planning; reviewed, and edited the final draft. MU wrote the original draft. AB, TK contributed to the original draft. OH and BM provided mentorship external to the core team, critically reviewed and edited drafts. NC and IB managed and supervised activity planning at local site. The author(s) read and approved the final manuscript.

Funding

Funding agencies had no role in the design and conduct of the study. The research team had full autonomy in all aspects of the study.

Availability of data and materials

Not applicable.

Ethics approval and consent to participate

Participants will be recruited at five local sites in Pakistan. The study has been approved by the National Bioethics Committee of Pakistan. All participants will provide written informed consent using a form approved by the local REB and will be advised of their right to withdraw from the study.

Consent for publication

Not applicable.

Competing interests

The authors declare that they have no competing interests.

References

Aboraya, A., Nasrallah, H. A., Elswick, D. E., Elshazly, A., Estephan, N., Aboraya, D., Berzingi, S., Chumbers, J., Berzingi, S., Justice, J., Zafar, J., & Dohar, S. (2018). Measurement-based care in psychiatry: Past, present, and future. *Innovations in Clinical Neuroscience*, *15*(11–12), 13–26.

Ahmed, B. S., Enam, S. F., Iqbal, Z., Murtaza, G., & Bashir, S. (2016). *Depression and anxiety: A snapshot of the situation in Pakistan*. https://doi.org/10.13189/IJNBS.2016.040202

Dowrick, C., Leydon, G. M., McBride, A., Howe, A., Burgess, H., Clarke, P., Maisey, S., & Kendrick, T. (2009). Patients' and doctors' views on depression severity questionnaires incentivised in UK quality and outcomes framework: Qualitative study. *BMJ*, *338*, b663. https://doi.org/10.1136/bmj.b663

Gadit, A. A. M., & Mugford, G. (2007). Prevalence of Depression among Households in Three Capital Cities of Pakistan: Need to Revise the Mental Health Policy. *PLOS ONE*, *2*(2), e209. https://doi.org/10.1371/journal.pone.0000209

Gelenberg, A. J. (2010). A Review of the Current Guidelines for Depression Treatment. *The Journal of Clinical Psychiatry*, *71*(7), 26478. https://doi.org/10.4088/JCP.9078tx1c

Guo, T., Xiang, Y.-T., Xiao, L., Hu, C.-Q., Chiu, H. F. K., Ungvari, G. S., Correll, C. U., Lai, K. Y. C., Feng, L., Geng, Y., Feng, Y., & Wang, G. (2015). Measurement-Based Care Versus Standard Care for Major Depression: A Randomized Controlled Trial With Blind Raters. *The American Journal of Psychiatry*, *172*(10), 1004–1013. https://doi.org/10.1176/appi.ajp.2015.14050652

Hamilton, M. (1960). A rating scale for depression. *Journal of Neurology, Neurosurgery, and Psychiatry*, *23*, 56–62. https://doi.org/10.1136/jnnp.23.1.56

Hong, R. H., Murphy, J. K., Michalak, E. E., Chakrabarty, T., Wang, Z., Parikh, S. V., Culpepper, L., Yatham, L. N., Lam, R. W., & Chen, J. (2021). <p>Implementing Measurement-Based Care for Depression: Practical Solutions for Psychiatrists and Primary Care Physicians</p>. *Neuropsychiatric Disease and Treatment*, *17*, 79–90. https://doi.org/10.2147/NDT.S283731

Karmaliani, R., Asad, N., Bann, C. M., Moss, N., McClure, E. M., Pasha, O., Wright, L. L., & Goldenberg, R. L. (2009). Prevalence of Anxiety, Depression and Associated Factors among Pregnant Women of Hyderabad, Pakistan. *The International Journal of Social Psychiatry*, *55*(5), 10.1177/0020764008094645. https://doi.org/10.1177/0020764008094645

Katon, W. J., Lin, E. H. B., Von Korff, M., Ciechanowski, P., Ludman, E. J., Young, B., Peterson, D., Rutter, C. M., McGregor, M., & McCulloch, D. (2010). Collaborative care for patients with depression

and chronic illnesses. *The New England Journal of Medicine*, *363*(27), Article 27. https://doi.org/10.1056/NEJMoa1003955

Kennedy, S. H., Lam, R. W., McIntyre, R. S., Tourjman, S. V., Bhat, V., Blier, P., Hasnain, M., Jollant, F., Levitt, A. J., MacQueen, G. M., McInerney, S. J., McIntosh, D., Milev, R. V., Muller, D. J., Parikh, S. V., Pearson, N. L., Ravindran, A. V., Uher, R., & Canmat Depression Work Group. (2016). Canadian Network for Mood and Anxiety Treatments (CANMAT) 2016 Clinical Guidelines for the Management of Adults with Major Depressive Disorder: Section 3. Pharmacological Treatments. *Can J Psychiatry*, *61*, 540–560. https://doi.org/10.1177/0706743716659417

Kessler, R. C., Berglund, P., Demler, O., Jin, R., Koretz, D., Merikangas, K. R., Rush, A. J., Walters, E. E., Wang, P. S., & National Comorbidity Survey, R. (2003). The epidemiology of major depressive disorder: Results from the National Comorbidity Survey Replication (NCS-R). *JAMA*, *289*, 3095–3105. https://doi.org/10.1001/jama.289.23.3095

Kessler, R., & Glasgow, R. E. (2011). A Proposal to Speed Translation of Healthcare Research Into Practice: Dramatic Change Is Needed. *American Journal of Preventive Medicine*, *40*(6), 637–644. https://doi.org/10.1016/j.amepre.2011.02.023

Liu, Q., He, H., Yang, J., Feng, X., Zhao, F., & Lyu, J. (2020). Changes in the global burden of depression from 1990 to 2017: Findings from the Global Burden of Disease study. *Journal of Psychiatric Research*, *126*, 134–140. https://doi.org/10.1016/j.jpsychires.2019.08.002

Ormel, J., Kessler, R. C., & Schoevers, R. (2019). Depression: More treatment but no drop in prevalence: how effective is treatment? And can we do better? *Current Opinion in Psychiatry*, *32*(4), 348–354. https://doi.org/10.1097/YCO.0000000000000505

Paxil -Highlights of Prescribing Medication. (2022). Suicidality and antidepressant drugs - food and drug ... (n.d.). Retrieved February 3, 2022, from https://www.accessdata.fda.gov/drugsatfda_docs/label/2012/020031s067,020710s031.pdf

Remeron -Highlights of Prescribing Medication. (2022, February). https://www.accessdata.fda.gov/drugsatfda_docs/label/2020/020415s029,%20021208s019lbl.pdf

Rush, A. J., Trivedi, M. H., Ibrahim, H. M., Carmody, T. J., Arnow, B., Klein, D. N., Markowitz, J. C., Ninan, P. T., Kornstein, S., Manber, R., Thase, M. E., Kocsis, J. H., & Keller, M. B. (2003). The 16-Item Quick Inventory of Depressive Symptomatology (QIDS), clinician rating (QIDS-C), and self-report (QIDS-SR): A psychometric evaluation in patients with chronic major depression. *Biological Psychiatry*, *54*(5), 573–583. https://doi.org/10.1016/s0006-3223(02)01866-8

Rush, A. J., Trivedi, M. H., Wisniewski, S. R., Nierenberg, A. A., Stewart, J. W., Warden, D., Niederehe, G., Thase, M. E., Lavori, P. W., Lebowitz, B. D., McGrath, P. J., Rosenbaum, J. F., Sackeim, H. A., Kupfer, D. J., Luther, J., & Fava, M. (2006). Acute and longer-term outcomes in depressed outpatients requiring one or several treatment steps: A STAR*D report. *The American Journal of Psychiatry*, *163*(11), 1905–1917. https://doi.org/10.1176/ajp.2006.163.11.1905

Sikander, S. (2020). Pakistan. *The Lancet Psychiatry*, *7*(10), 845. https://doi.org/10.1016/S2215-0366(20)30387-4

Trivedi, M. H. (2009). Tools and strategies for ongoing assessment of depression: A measurement-based approach to remission. *The Journal of Clinical Psychiatry*, *70 Suppl 6*, 26–31. https://doi.org/10.4088/JCP.8133su1c.04

Trivedi, M. H., & Daly, E. J. (2007). Measurement-based care for refractory depression: A clinical decision support model for clinical research and practice. *Drug & Alcohol Dependence*, *88*, S61–S71. https://doi.org/10.1016/j.drugalcdep.2007.01.007

Trivedi, M. H., Fava, M., Wisniewski, S. R., Thase, M. E., Quitkin, F., Warden, D., Ritz, L., Nierenberg, A. A., Lebowitz, B. D., Biggs, M. M., Luther, J. F., Shores-Wilson, K., & Rush, A. J. (2006). Medication augmentation after the failure of SSRIs for depression. *N Engl J Med*, *354*, 1243–1252. https://doi.org/354/12/1243 [pii] 10.1056/NEJMoa052964

Trivedi, M. H., Papakostas, G. I., Jackson, W. C., & Rafeyan, R. (2020). Implementing Measurement-Based Care to Determine and Treat Inadequate Response. *The Journal of Clinical Psychiatry*, *81*(3), 26563. https://doi.org/10.4088/JCP.OT19037BR1

Trivedi, M. H., Rush, A. J., Crismon, M. L., Kashner, T. M., Toprac, M. G., Carmody, T. J., Key, T., Biggs, M. M., Shores-Wilson, K., Witte, B., Suppes, T., Miller, A. L., Altshuler, K. Z., & Shon, S. P. (2004). Clinical Results for Patients With Major Depressive Disorder in the Texas Medication Algorithm Project. *Archives of General Psychiatry*, *61*(7), 669–680. https://doi.org/10.1001/archpsyc.61.7.669

Trivedi, M. H., Rush, A. J., Wisniewski, S. R., Nierenberg, A. A., Warden, D., Ritz, L., Norquist, G., Howland, R. H., Lebowitz, B., McGrath, P. J., Shores-Wilson, K., Biggs, M. M., Balasubramani, G. K., Fava, M., & Star D. Study Team. (2006). Evaluation of outcomes with citalopram for depression using measurement-based care in STAR*D: implications for clinical practice. *Am J Psychiatry*, *163*, 28–40. https://doi.org/10.1176/appi.ajp.163.1.28

Vos, T., Lim, S. S., Abbafati, C., Abbas, K. M., Abbasi, M., Abbasifard, M., Abbasi-Kangevari, M., Abbastabar, H., Abd-Allah, F., Abdelalim, A., Abdollahi, M., Abdollahpour, I., Abolhassani, H., Aboyans, V., Abrams, E. M., Abreu, L. G., Abrigo, M. R. M., Abu-Raddad, L. J., Abushouk, A. I., … Murray, C. J. L. (2020). Global burden of 369 diseases and injuries in 204 countries and territories, 1990–2019: A systematic analysis for the Global Burden of Disease Study 2019. *The Lancet*, *396*(10258), 1204–1222. https://doi.org/10.1016/S0140-6736(20)30925-9

Wisniewski, S. R., Rush, A. J., Balasubramani, G. K., Trivedi, M. H., Nierenberg, A. A., & STARD Investigators. (2006). Self-rated global measure of the frequency, intensity, and burden of side effects. *Journal of Psychiatric Practice*, *12*(2), 71–79. https://doi.org/10.1097/00131746-200603000-00002

Xiao, L., Qi, H., Zheng, W., Xiang, Y.-T., Carmody, T. J., Mayes, T. L., Trivedi, M. H., & Wang, G. (2021). The effectiveness of enhanced evidence-based care for depressive disorders: A meta-analysis of randomized controlled trials. *Translational Psychiatry*, *11*(1), 1–8. https://doi.org/10.1038/s41398-021-01638-7

Young, R. C., Biggs, J. T., Ziegler, V. E., & Meyer, D. A. (1978). A rating scale for mania: Reliability, validity and sensitivity. *The British Journal of Psychiatry: The Journal of Mental Science*, *133*, 429–435. https://doi.org/10.1192/bjp.133.5.429

Zhu, M., Hong, R. H., Yang, T., Yang, X., Wang, X., Liu, J., Murphy, J. K., Michalak, E. E., Wang, Z., Yatham, L. N., Chen, J., & Lam, R. W. (2021). The Efficacy of Measurement-Based Care for Depressive Disorders: Systematic Review and Meta-Analysis of Randomized Controlled Trials. *The Journal of Clinical Psychiatry*, *82*(5), 37090. https://doi.org/10.4088/JCP.21r14034

Statistical Analysis Plan (SAP) for the MBC in Pakistan Study

Principal Investigator: Dr. M. Ishrat Husain

Version: 1.0

Creation Date: April 10, 2022

Final Sign-off Date: November 14, 2024

The study is based on the published study done by Guo et al (2015) in China. Thus, the protocol (submitted to the National Bioethics Committee in Pakistan on April 10, 2022) and this SAP are entirely based on the protocol and data analysis section of Guo et al, 2015.

Contents

1. Specific Aims and Hypotheses

2. Outcomes

3. Statistical Methods

3.1. Interim Analyses

3.2. Statistical Analyses

1. Specific Aims and Hypotheses

The aim of this trial is to determine the efficacy and safety of MBC in patients with MDD in comparison with standard care in Pakistan. To reduce the variance found in treatment-as-usual and isolate the impact of MBC, this trial will limit medication choices to either paroxetine or mirtazapine (as per previously published trials). We hypothesize that, compared to standard care group, the MBC group will have significantly higher rates and shorter times to response and remission, along with lower rates of dropout and side effect burden.

2. Outcomes

A form will be designed to collect sociodemographic and clinical characteristics from the medical records, and this information will be confirmed in a clinical interview. HDRS scores will be used to inform two primary outcomes measures: estimated time from randomization to response (defining response as ≥ 50% reduction in HDRS from baseline) and remission (defined as HDRS score ≤ 7) (Hamilton, 1960).

Secondary outcome measures will include changes in severity of depressive symptoms (HDRS score). An additional checklist with six common side effects (dry mouth, diarrhea or constipation, dizziness or drowsiness, loss of appetite or nausea, headache, and excessive sweating); and the FISBER, will be used to measure side effects at each treatment visit. The pill count method will be used to measure treatment adherence.

3. Statistical Methods

3.1. Interim Analyses

The protocol stated that a treatment-blind statistician may carry out interim quality checks and efficacy analysis. No interim analyses were needed, and none were done.

3.2. Statistical Analyses

A treatment-blind statistician will analyze the data after completion of follow-ups for all recruited participants using SPSS for Windows, version 20.0 (IBMCorp., Armonk, N.Y.).

Like the Guo et al. (2015) trial, we will use independent-sample t-tests, Mann-Whitney U tests, and chi-square tests, to compare sociodemographic and clinical characteristics, discontinuation, response and remission rates, and side effects as appropriate. Full intent-to-treat analyses will be carried out to determine all-cause discontinuation. Estimated time from randomization to response and remission will be calculated using Kaplan-Meier survival analyses, and a comparative analysis will be facilitated using the Cox proportional hazards regression model. Analysis of covariance will be used to compare the changes in HDRS scores between the two groups. Additional sub-group analysis might be carried out to understand group-differences due to age, gender, duration of illness, and number of previous episodes. The significance threshold will be $p<0.05$ (two-tailed) for all analyses.

References:

1. Guo, T., Xiang, Y.-T., Xiao, L., Hu, C.-Q., Chiu, H. F. K., Ungvari, G. S., Correll, C. U., Lai, K. Y. C., Feng, L., Geng, Y., Feng, Y., & Wang, G. (2015). Measurement-Based Care Versus Standard Care for Major Depression: A Randomized Controlled Trial With Blind Raters. *The American Journal of Psychiatry*, *172*(10), 1004–1013. https://doi.org/10.1176/appi.ajp.2015.14050652

2. Hamilton, M. (1960). A rating scale for depression. *Journal of Neurology, Neurosurgery, and Psychiatry*, *23*, 56–62. https://doi.org/10.1136/jnnp.23.1.56
