# Supplementary material for: Measurement-Based Care to Enhance Antidepressant Treatment Outcomes in Major Depressive Disorder: A Randomized Clinical Trial
Source: JAMA Netw Open. 2025 Sep 2;8(9):e2529427. doi: 10.1001/jamanetworkopen.2025.29427 (PMC12406064; doi:10.1001/jamanetworkopen.2025.29427)
Supplement: Supplement 2. — eTable 1. The Measurement-Based Care Schedule eTable 2. Medication Adherence as Assessed via Percentage of Pills Consumed at Each Time Point eTable 3. Adverse Effects Among Participants Receiving Measurement-Based Care or Standard Care eFigure. Estimated Median Time to Discontinuation by Kaplan-Meier Analysis [file jamanetwopen-e2529427-s002.pdf]

Supplemental Online Content

Husain MI, Nigah Z, Ansari SUH, et al. Measurement-based care to enhance antidepressant treatment outcomes in major depressive disorder: a randomized clinical trial. *JAMA Netw Open*. 2025;8(9):e2529427. doi:10.1001/jamanetworkopen.2025.29427

eTable 1. The Measurement-Based Care Schedule

eTable 2. Medication Adherence as Assessed via Percentage of Pills Consumed at Each Time Point

eTable 3. Adverse Effects Among Participants Receiving Measurement-Based Care or Standard Care

eFigure. Estimated Median Time to Discontinuation by Kaplan-Meier Analysis

This supplemental material has been provided by the authors to give readers additional information about their work.

© 2025 Husain MI et al. *JAMA Network Open*

eTable 2. The Measurement-Based Care Schedule

Time Point and Measure	Clinical Status	Treatment Plan	
		Paroxetine	Mirtazapine
**Week 0**		Start at 10-20 mg/day	Start at 7.5-15 mg/day, increase to 15 mg/day by week 1
**Week 2**			
Outpatient visit,		Increase dose to 20mg/day	Increase dose to 30 mg/day
**Week 4**			
QIDS-SR score, ≤5	Remission	Continue current dose	Continue current dose
QIDS-SR score, 6–8	Partial response	Continue current dose or consider increasing to 30 mg/day	Continue current dose or consider increasing to 45 mg/day
	SEs intolerable	Continue current dose and address SEs, or switch to mirtazapine	Continue current dose and address SEs, or switch to paroxetine
QIDS-SR score, ≥9	Nonresponse	Increase to 30 mg/day or switch to mirtazapine	Increase to 45 mg/day or switch to paroxetine
	SEs intolerable	Switch to mirtazapine	Switch to paroxetine
**Week 6**			
QIDS-SR score, ≤5	Remission	Continue current dose	Continue current dose
QIDS-SR score, 6–8	Partial response	Continue current dose or consider increasing to 40 mg/day	Continue current dose or consider increasing to 45 mg/day
	SEs intolerable	Continue current dose and address SEs, or switch to mirtazapine	Continue current dose and address SEs, or switch to paroxetine
QIDS-SR score, ≥9	Nonresponse	Increase to 40 mg/day	Increase to 45 mg/day or switch to paroxetine
	SEs intolerable	Switch to mirtazapine	Switch to paroxetine
**Week 8**			
QIDS-SR score, ≤5	Remission	Continue current dose	Continue current dose
QIDS-SR score, 6–8	Partial response	Continue current dose or consider increasing to 50 mg/day	Continue current dose or consider increasing to 45 mg/day
	SEs intolerable	Switch to mirtazapine	Switch to paroxetine
QIDS-SR score, ≥9	Nonresponse	Switch to mirtazapine	Switch to paroxetine
	SEs intolerable	Switch to mirtazapine	Switch to paroxetine
**Week 10**			
QIDS-SR score, ≤5	Remission	Continue current dose	Continue current dose
QIDS-SR score, 6–8	Partial response	Continue current dose or consider increasing to 60 mg/day	Continue current dose or consider switch to paroxetine
	SEs intolerable	Switch to mirtazapine	Switch to paroxetine
QIDS-SR score, ≥9	Nonresponse	Switch to mirtazapine	Switch to paroxetine
	SEs intolerable	Switch to mirtazapine	Switch to paroxetine
**Week 12**			
QIDS-SR score, ≤5	Remission	Continue current dose and follow up	Continue current dose and follow up

© 2025 Husain MI et al. *JAMA Network Open*

Time Point and Measure	Clinical Status	Treatment Plan	
		Paroxetine	Mirtazapine
QIDS-SR score, 6–8	Partial response	Continue current dose and follow up, or consider switch to mirtazapine	Continue current dose and follow up, or consider switch to paroxetine
	SEs intolerable	Switch to mirtazapine	Switch to paroxetine
QIDS-SR score, ≥9	Nonresponse	Switch to mirtazapine	Switch to paroxetine
	SEs intolerable	Switch to mirtazapine	Switch to paroxetine

© 2025 Husain MI et al. *JAMA Network Open*

eTable 2. Medication adherence as assessed via percentage of pills consumed at each time point

	Measurement-Based Care Group		Standard Treatment Group			
	**No.**	**Mean %(SD)**	**No.**	**Mean %(SD)**	**Mean Diff. (%)**	**P-value**
**Week-2**	68	97.5 (5.6)	72	98.6 (5.2)	-1.1 (-2.9 – 0.7)	.24
**Week-4**	66	98.2 (4.8)	71	99.3 (2.4)	-1.1 (-2.4 – 0.1)	.08
**Week-6**	64	96.2 (7.0)	69	98.7 (4.6)	-2.4 (-4.5 - -0.4)	.020
**Week-8**	64	96.9 (6.2)	69	98.5 (5.6)	-1.6 (-3.6 – 0.4)	.120
**Week-10**	64	96.6 (7.0)	68	98.2 (5.5)	-1.6 (-3.7 – 0.6)	.16
**Week-12**	64	97.4 (5.3)	68	98.9 (3.1)	-1.5 (-3.0 to – -0.1)	.048
**Week-24**	64	96.1 (9.1)	67	98.1 (6.2)	-1.9 (-4.6 – 0.8)	.15

© 2025 Husain MI et al. *JAMA Network Open*

eTable 3. Adverse Effects Among Participants Receiving Measurement-Based Care or Standard Care

Adverse Effect	Measurement-Based Care Group	Standard Care Group	p-value
Dry mouth	9 of 64 (14.1%)	19 of 67 (28.4%)	.056
Diarrhea	5 of 64 (7.8%)	3 of 67 (4.5%)	.486
Constipation	2 of 64 (3.1%)	3 of 67 (4.5%)	.999
Dizziness or drowsiness	12 of 64 (18.8%)	14 of 67 (20.9%)	.828
Loss of appetite	6 of 64 (9.4%)	14 of 67 (20.9%)	.089
Nausea	7 of 64 (10.9%)	11 of 67 (16.4%)	.450
Headache	15 of 64 (23.4%)	25 of 67 (37.3%)	.092
Excessive sweating	6 of 64 (9.4%)	4 of 67 (6.0%)	.525

eFigure. Estimated Median Time to Discontinuation by Kaplan-Meier Analysis

Measure	Measurement-Based Care Group n=64	Standard Care Group n=68	p-value
Estimated time to remission, median (IQR), weeks	2 [2-4]	4 [2-6]	0.44

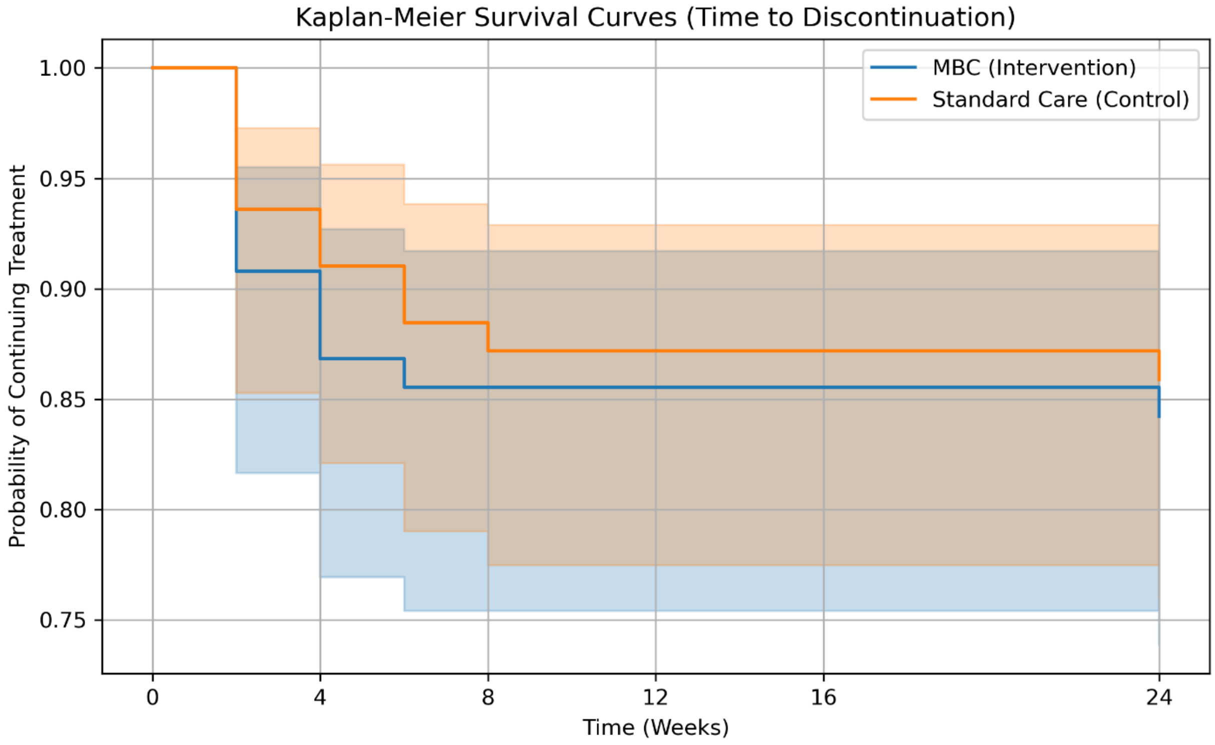

© 2025 Husain MI et al. *JAMA Network Open*
