# Supplementary material for: Measurement-Based Care to Enhance Antidepressant Treatment Outcomes in Major Depressive Disorder: A Randomized Clinical Trial
Source: JAMA Netw Open. 2025 Sep 2;8(9):e2529427. doi: 10.1001/jamanetworkopen.2025.29427 (PMC12406064; doi:10.1001/jamanetworkopen.2025.29427)
Supplement: Supplement 3. — Data Sharing Statement [file jamanetwopen-e2529427-s003.pdf]

Data Sharing Statement

Data

Additional Information: ClinicalTrials.gov: NCT05431374
Data available: Yes
Data types: Deidentified participant data
How to access data: ishrat.husain@camh.ca
When available: With publication

Supporting Documents

Document types: Informed consent form
How to access documents: ishrat.husain@camh.ca
When available: With publication

Additional Information

Who can access the data: Researchers whose proposed use of the data has been approved
Types of analyses: Specified purposes.
Mechanisms of data availability: After approval of a proposal.
